# Screen Time and Myopia-Related Outcomes in European Children: A Systematic Review

**DOI:** 10.3390/vision10030043

**Published:** 2026-07-13

**Authors:** Hammaad Khalid, Ali Istanaksai, Mishelle Abbasi

**Affiliations:** 1 Medicine, Leeds Teaching Hospitals NHS Trust, Leeds LS1 3EX, UK; 2 Medicine, North Middlesex University Hospital, London N18 1QX, UK; 3 Paediatrics, Airedale Hospital, Keighley BD20 6TD, UK

**Keywords:** paediatric ophthalmology, refractive error, European children, outdoor exposure, outdoor activity, smartphone use, near work, digital device use, screen time, paediatric myopia

## Abstract

Myopia is an increasingly common refractive disorder in children, with growing concern regarding the potential role of screen time and digital device use in its development and progression. This systematic review assessed the association between screen-related behaviours and myopia in European children and adolescents. A systematic search of Ovid Embase, Ovid MEDLINE, Scopus, Web of Science and Cochrane/CENTRAL was conducted in accordance with PRISMA guidance. Original English-language studies published within the last 10 years were included if they involved participants aged <18 years, were conducted in Europe, and investigated screen time, digital device use, near-work behaviour or related lifestyle exposures in relation to myopia-related outcomes. A total of 1196 records were identified across the databases. After duplicate removal, title and abstract screening, and full-text assessment, 14 studies were included. Overall, the evidence suggested an association between screen-related behaviours, prolonged near work, reduced outdoor exposure and myopia-related outcomes in European children. However, findings were heterogeneous, and screen exposure was often measured using broad or proxy variables, including total screen time, smartphone use, internet data consumption, computer use, handheld near-screen use and COVID-19-related lifestyle change. Outdoor activity appeared to be a protective factor, while parental myopia was an important risk factor and confounder. Most included studies were observational, many were cross-sectional, and several relied on self- or parent-reported exposure measures, limiting causal inference and introducing potential recall bias. Overall, the certainty of evidence was low to very low. Practical advice should therefore be framed around balanced digital device use, regular breaks from prolonged near work, appropriate viewing distances and increased outdoor activity, while further longitudinal studies using objective screen-use measures, cycloplegic refraction and axial length outcomes are needed to clarify causality.

## 1. Introduction

Myopia (near-sightedness) is a refractive error in which light focuses anterior to the retina when accommodation is relaxed, resulting in blurred distance vision. It is commonly caused by excessive axial elongation of the eyeball and/or increased refractive power of the cornea and crystalline lens [1]. Myopia is typically defined as a spherical equivalent refractive error of ≤−0.50 dioptres (D) [2].

High myopia, defined by the International Myopia Institute (IMI) as ≤−6.0 D or an axial length >26 mm, is associated with an increased risk of retinal detachment, myopic maculopathy, glaucoma, and cataract [1,2,3]. The prevalence of myopia has increased substantially worldwide, particularly among children and adolescents, and is projected to affect approximately 50% of the global population by 2050 [1]. Consequently, the World Health Organisation (WHO) has recognised myopia as a major public health challenge [4,5]. Since the COVID-19 pandemic, screen exposure has increased substantially owing to the widespread adoption of remote learning and the growing integration of digital technology into children’s education and daily lives [6,7]. One proposed mechanism linking screen exposure to myopia progression is the displacement of outdoor activity and increased engagement in near-work tasks, which may reduce retinal dopamine release and promote axial elongation [8,9,10,11]. However, evidence from European paediatric populations remains limited, as most studies investigating the association between screen exposure and myopia have been conducted in Asia, where myopia prevalence is considerably higher, reaching 80–90% in some adolescent populations [9,12,13]. In contrast, childhood myopia prevalence in Europe has been estimated at approximately 7.15% [14]. Variations in prevalence, educational demands, near-work intensity, urbanisation, and outdoor activity may influence myopia risk and limit the generalisability of findings from Asian populations to European children.

In this review, “screen exposure” is used as an umbrella term for several related digital and near-work behaviours, including smartphone use, tablet use, computer use, television viewing, internet data use and broader screen-based near work. It should not be interpreted as a single biologically uniform exposure. Rather, screen exposure may act as a surrogate for multiple behaviours that could influence myopia risk, including prolonged near work, shorter viewing distance, sustained accommodation, reduced breaks, increased indoor time, reduced outdoor exposure and educational demands. This conceptual limitation was considered when interpreting the findings.

The decision to focus on European populations was made to provide a region-specific synthesis that may be more applicable to European clinical, educational and public health settings. Differences in baseline myopia prevalence, ethnicity, educational intensity, urbanisation, school routines, outdoor activity and digital-device behaviours may limit the direct generalisability of findings from high-prevalence Asian populations to European children. However, this geographical restriction also narrows the evidence base and may limit external validity. The findings of this review should therefore be interpreted as complementary to, rather than a replacement for, wider international systematic reviews. Accordingly, this systematic review aimed to evaluate the association between digital screen exposure and myopia in European children and adolescents.

## 2. Methods

**Figure 1:**
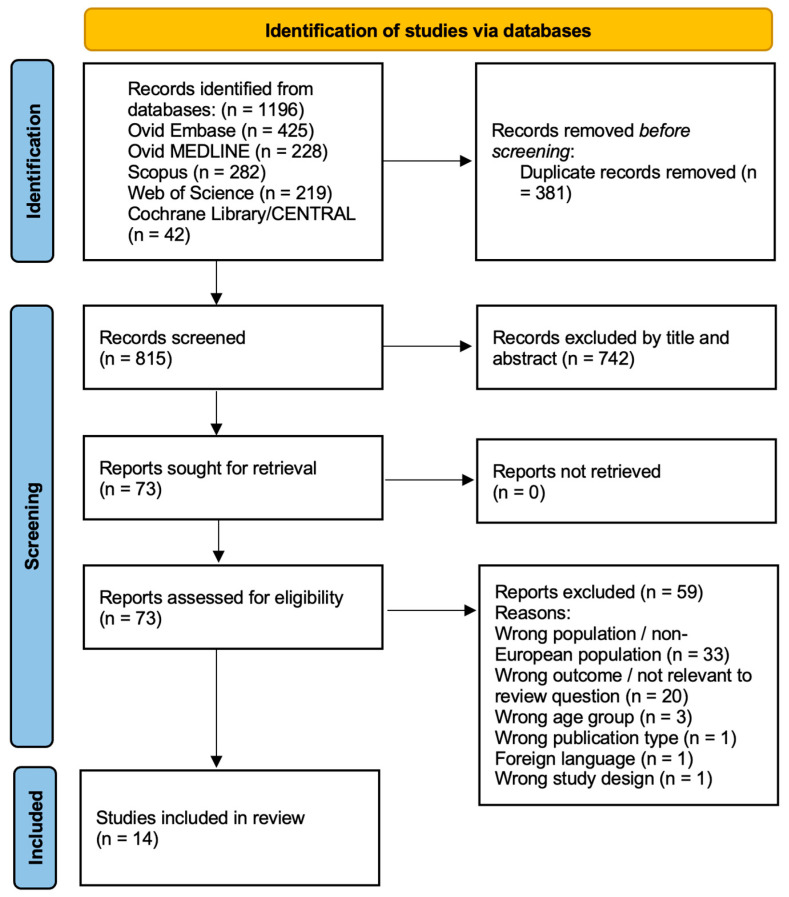
PRISMA 2020 flow diagram of the updated study selection process, including searches of Ovid Embase, Ovid MEDLINE, Scopus, Web of Science and Cochrane Library/CENTRAL. (PRISMA: Preferred Reporting Items for Systematic Reviews and Meta-Analyses).

This systematic review was conducted in accordance with the Preferred Reporting Items for Systematic Reviews and Meta-Analyses (PRISMA) 2020 guidelines [15] (Figure 1). A completed PRISMA 2020 checklist is provided as Supplementary Table S1. The review protocol was not prospectively registered.

### 2.1. Search Strategy

A systematic literature search was initially conducted in January 2026 using Ovid Embase and Ovid MEDLINE. The search was subsequently expanded to include Scopus, Web of Science and the Cochrane Library/CENTRAL. The updated search was conducted in July 2026 using search terms adapted from the original strategy. Search terms relating to myopia were combined with terms relating to screen exposure, digital device use and paediatric populations using the Boolean operators “AND” and “OR”. The search strategy included terms such as “myop*”, “near-sightedness”, “short-sightedness”, “refractive error”, “screen time”, “smartphone”, “tablet”, “digital device”, “computer use”, “adolescent”, “teenager”, “schoolchildren”, “child”, “paediatric” and “pediatric”. The asterisk (*) was used as a truncation symbol to search for all words sharing the root ‘myop-’ with variable endings. Searches were limited to English-language studies published between 2016 and 2026 involving participants aged under 18 years, where database filters allowed. The full search strategy is provided in Appendix B.

A total of 1196 records were identified across Ovid Embase, Ovid MEDLINE, Scopus, Web of Science and Cochrane Library/CENTRAL. After duplicate removal, 815 records remained for title and abstract screening. Seventy-three reports were assessed for eligibility at full-text review, and 14 studies were included in the final review. Reference lists of relevant articles and systematic reviews were also manually screened to identify any additional eligible studies. The review question and eligibility criteria were structured using a Population, Intervention/Exposure, Comparison and Outcome (PICO) framework. The full PICO framework is provided in Appendix A.

### 2.2. Eligibility Criteria

Studies were eligible for inclusion if they were original research studies involving children or adolescents aged under 18 years, were conducted in European populations, and investigated the association between screen time or digital device use and myopia-related outcomes. Eligible exposures included screen time, smartphone use, tablet use, computer use, handheld digital device use and broader digital near-work behaviours. Eligible outcomes included myopia prevalence, incidence or progression, refractive error, spherical equivalent, axial elongation or other reported myopia-related visual outcomes.

Studies were excluded if they were not conducted in European populations, included adult-only populations, did not assess screen exposure or digital device use, did not report a myopia-related outcome, were not original research articles, were not published in English, or were published outside the predefined 10-year search period.

### 2.3. Study Selection

Following duplicate removal, titles and abstracts were screened independently by two reviewers against the predefined eligibility criteria. Full texts of potentially eligible studies were then assessed independently by the same reviewers. Disagreements regarding study inclusion were resolved through discussion and consensus. The study selection process is summarised using a PRISMA 2020 flow diagram (Figure 1).

### 2.4. Data Extraction

Data were extracted using a standardised data extraction approach. Extracted variables included author, year of publication, country, study design, sample size, participant age group, exposure type, myopia-related outcome measures, key findings and relevant confounding factors such as outdoor activity, physical activity and parental myopia. Where available, reported effect estimates, *p*-values and measures of refractive change were extracted.

### 2.5. Risk of Bias Assessment

Risk of bias was assessed using study design-specific appraisal tools. Cohort studies were assessed using the Newcastle–Ottawa Scale (NOS), with results presented in Table 1. Cross-sectional studies were assessed using the Joanna Briggs Institute (JBI) Critical Appraisal Checklist for analytical cross-sectional studies, with results presented in Table 2. Risk of bias assessments were performed independently by two reviewers, with disagreements resolved through discussion and consensus.

Risk-of-bias assessments were used to inform the narrative synthesis, with greater caution applied to findings from cross-sectional studies, studies using proxy or self-reported exposure measures, and studies with incomplete adjustment for important confounders.

### 2.6. Data Synthesis

The characteristics and main findings of the included studies are summarised in Table 3. Due to heterogeneity in study design, exposure definitions, outcome measures, methods of myopia assessment and adjustment for confounders, meta-analysis was not performed. The main sources of heterogeneity were considered before deciding on narrative synthesis. Exposure definitions varied substantially across studies and included total screen time, smartphone use, internet data consumption, computer use, handheld near-screen use, near-work behaviours and COVID-19-related lifestyle change. Outcome measures also differed, including myopia prevalence, refractive error, spherical equivalent, refractive shift, axial length and myopia progression. In addition, included studies assessed different age groups and varied in their adjustment for important confounders such as outdoor activity, parental myopia, socioeconomic status, educational demands and baseline refractive status. As a result, pooled effect estimates were considered unlikely to be clinically meaningful.

Findings were therefore synthesised narratively. Studies were grouped and interpreted according to key themes, including overall screen exposure, device type, near-work behaviour, outdoor activity, parental myopia, lifestyle factors and COVID-19-related changes in visual behaviour. Attention was given to whether studies assessed screen exposure using direct measures or through related proxy behaviours such as near work, reduced outdoor activity or increased indoor time. Risk-of-bias assessments were considered when interpreting the findings, with greater caution applied to cross-sectional studies, proxy exposure measures, self- or parent-reported exposure data and studies with incomplete adjustment for confounding factors.

### 2.7. Certainty of Evidence

A formal GRADE assessment was considered; however, due to substantial heterogeneity in study design, exposure definitions, outcome measures and methods of confounder adjustment, a full outcome-specific GRADE assessment was not performed. Instead, the overall certainty of evidence was considered narratively using key GRADE domains, including risk of bias, inconsistency, indirectness, imprecision and publication bias.

Overall, the certainty of evidence was judged to be low to very low. This was mainly due to the predominance of observational and cross-sectional study designs, reliance on self- or parent-reported exposure measures, heterogeneity in definitions of screen exposure, variation in myopia outcome assessment and inconsistent adjustment for important confounders such as outdoor activity, parental myopia, socioeconomic status and educational demands. Therefore, the findings should be interpreted as evidence of association rather than causation.

## 3. Review

### 3.1. Screen Exposure and Myopia

Across the 14 included studies, evidence generally suggested an association between screen-related behaviours, near-work exposure, reduced outdoor activity and myopia-related outcomes in European paediatric populations [16,17,18,19,20,21,22,23,24,25,26,27,28,29]. However, the strength and consistency of this association varied substantially across studies because of differences in study design, exposure definitions, outcome assessment and adjustment for confounders. Importantly, several studies measured proxy or composite exposures, such as smartphone internet data, total screen time, computer use, near-work behaviour or COVID-19-related lifestyle change, rather than screen exposure as a single independent biological exposure. Therefore, the findings should be interpreted as evidence of association within a broader visual and behavioural environment, rather than as proof that screen exposure alone causes myopia.

McCrann et al. conducted a cross-sectional study in Ireland involving 402 participants and found that myopic children used approximately twice as much smartphone internet data as non-myopic children (1130.71 ± 1748.14 MB/day vs. 613.63 ± 902.15 MB/day, *p* = 0.001) [20]. These findings suggest a positive association between smartphone use and childhood myopia. A notable strength of the study was the use of objectively measured internet data, reducing reliance on self-reported screen exposure. However, internet data usage is only a proxy measure and does not capture important factors such as viewing distance, screen duration, device type, near-work intensity, posture, lighting conditions, or frequency of breaks. Furthermore, the large standard deviations indicate substantial variability in usage patterns between participants. Consequently, although the study supports an association between smartphone use and myopia, it does not establish causality or provide insight into the underlying mechanisms. In another cross-sectional study of 7497 Spanish children, Alvarez-Peregrina et al. found that myopic children reported significantly greater screen exposure and lower levels of outdoor activity than their non-myopic peers (*p* < 0.01) [22]. This finding is supported by an Irish cross-sectional study conducted in 2019 involving 1626 participants, which found that over half of myopic children exceeded two hours of daily screen time, compared with approximately one-third of the overall population [23]. Harrington and O’Dwyer similarly reported that higher screen time was associated with more myopic refraction and increased odds of myopia in Irish children aged 6–7 years [24].

The Madrid school-based study provided further European evidence but suggested a more cautious interpretation of screen time. In this cross-sectional study of 2489 Spanish primary school children using cycloplegic refraction, myopia prevalence was 6.5% in second-grade children and 18.7% in sixth-grade children [29]. Although children with myopia were more frequently observed in higher screen-time categories, screen time was not independently associated with myopia after multivariate adjustment. Instead, parental myopia remained an important risk factor, while outdoor activity, particularly during weekends, was associated with lower myopia prevalence. This supports the interpretation that screen time may partly act as a proxy for broader lifestyle and visual behaviours rather than an isolated independent exposure.

Evidence from cohort studies further supports an association between visual environmental exposures and myopia. In the Generation R cohort, greater combined near-work exposure was associated with increased odds of myopia (OR 1.072, 95% CI 1.047–1.098) among 5074 participants [16]. Similarly, the Myopia App Study assessed smartphone use via an application. It found that use of the device for 3.7 to 3.8 h per day and continuous use of more than 20 min was linked with more myopic refraction [19]. Hansen et al. reported that Danish adolescents with more than six hours of daily screen use had approximately twice the risk of myopia, with an overall myopia prevalence of around 25% [17]. These findings suggest that the association between screen exposure and myopia may be more pronounced at higher levels of exposure or when screen use occurs alongside other near-work activities.

Although several studies reported associations between screen-related behaviours and myopia-related outcomes, their predominantly observational design precludes conclusions regarding causality. It remains unclear whether increased screen exposure contributes to myopia development, whether children with myopia are more likely to engage in screen-based activities, or whether both are influenced by shared factors such as outdoor activity, educational demands and near-work intensity. Residual confounding, therefore, cannot be excluded.

Other included studies examined related lifestyle and environmental factors, including parental influence, outdoor activity, broader lifestyle behaviours and pandemic-related changes to daily routines. Iyer et al. assessed handheld near-screen use and parental influence, reporting that 26.0% of children used near-screens for more than two hours per day and that parents with myopia had greater awareness and were more likely to reduce their children’s screen time [25]. Sánchez-Tena et al. similarly examined parental myopia, outdoor activity and lifestyle factors, reporting a myopia prevalence of 12.7% and identifying outdoor activity as protective [28]. Schuster et al. reported increasing myopia prevalence over time among 17,640 German children and adolescents [19], while Rudnicka et al. demonstrated global variation and rising childhood myopia trends, with near work and reduced outdoor exposure implicated as potential contributors [27]. Collectively, these studies suggest that screen exposure is embedded within broader behavioural and environmental patterns, including near work, indoor activity and reduced outdoor exposure. The observed associations therefore reflect multifactorial risk rather than screen use alone.

Two studies examined myopia-related outcomes within the context of the COVID-19 pandemic. These studies suggest that broader changes in children’s visual environment may influence refractive outcomes, but they should not be interpreted as evidence that screen exposure alone caused myopic progression. During lockdown, several behaviours changed simultaneously, including remote learning, near-work duration, outdoor activity, physical activity, sleep patterns and daily routines. The Italian study reported that refractive error was stable before the pandemic but shifted significantly towards myopia after lockdown, while Alvarez-Peregrina et al. reported a significant myopic shift after COVID-19 home confinement [18,26]. These findings support the importance of broader lifestyle and environmental change, but attribution to screen exposure alone is not appropriate.

Overall, the included studies support an association between screen-related behaviours, near-work behaviour and childhood myopia-related outcomes, but do not establish causation. Evidence came from studies assessing computer use, screen-device duration, smartphone data, app-recorded smartphone use, general screen time, COVID-19-related lifestyle change, parental influence, outdoor activity, population trends and broader environmental factors. Across the evidence base, screen exposure was rarely isolated from prolonged near work, increased indoor activity and reduced outdoor exposure. Therefore, screen-related behaviours may contribute to myopia risk as part of a wider behavioural and visual environment, rather than acting as an independent causal factor. Further longitudinal studies using standardised exposure definitions, objective screen-use measures and adequate adjustment for outdoor activity and parental myopia are required.

### 3.2. Device Type and Near-Work

The relationship between screen exposure and childhood myopia appears to be influenced by the type of device used and the nature of visual behaviour. The term “screen time” encompasses activities with different visual demands, as smartphones, tablets, computers and televisions vary in viewing distance, duration of continuous use, posture and accommodative demand. Consequently, studies using broad measures of total screen time may dilute device-specific effects and partly explain differences in the strength of observed associations.

The most device-specific evidence came from studies assessing handheld digital devices. McCrann et al. objectively measured smartphone internet data in 402 Irish children and found that myopic participants used significantly more smartphone data than non-myopic children (1130.71 ± 1748.14 MB/day vs. 613.63 ± 902.15 MB/day, *p* = 0.001) [20]. Similarly, the Myopia App Study used smartphone application-based monitoring and reported average smartphone use of approximately 3.7–3.8 h/day, with continuous use of ≥20 min associated with more myopic refractive error, particularly among adolescents with lower outdoor exposure [21]. Iyer et al. provided further device-type context, reporting that 26.0% of children used handheld near-screen devices for more than two hours/day, and that parents with myopia were more likely to reduce their children’s screen time [25]. Overall, these studies suggest that handheld device use may be particularly relevant to myopia-related near-work exposure, especially when use is prolonged and uninterrupted. The strength of this evidence lies in its device-specific focus and, in McCrann et al. and the Myopia App Study, the use of objective digital monitoring rather than self-reported screen time. However, the evidence remains observational, and these measures do not fully capture viewing distance, posture and visual breaks.

In contrast, several studies assessed broader screen exposure without separating individual device types. The Generation R cohort assessed computer use as part of combined near-work exposure, alongside other visually demanding activities such as reading and other close-work tasks. This aggregated definition of exposure, rather than isolating smartphone or tablet use, was associated with increased odds of myopia (OR 1.072, 95% CI 1.047–1.098) among 5074 participants [16]. The Danish study measured overall daily screen-device use (including television, computers, tablets and smartphones combined) and found that adolescents with more than six hours of daily screen exposure had approximately twice the risk of myopia [17]. Similarly, Alvarez-Peregrina’s Spanish study and both studies from Ireland assessed general screen time pooled across multiple devices. They reported significantly greater overall screen exposure among myopic children compared with non-myopic peers and found that screen time was associated with more myopic refraction and increased odds of myopia in younger children aged 6–7 years [22,23,24]. However, across these studies, screen exposure was consistently treated as a composite measure encompassing multiple devices (smartphones, tablets, computers and television). As a result, it is not possible to determine whether the observed associations were driven predominantly by handheld near-screen use, longer-distance screen viewing, or broader indoor near-work and lifestyle behaviours.

Although handheld devices may be viewed at shorter distances and may involve sustained accommodation, the current evidence does not allow firm conclusions about whether smartphones or handheld devices have a stronger independent association with myopia than other screen modalities. Across the included studies, device type was often mixed with other behaviours, including prolonged near work, reduced outdoor exposure, educational screen use and indoor activity. Therefore, differences between smartphones, tablets, computers and television should be interpreted cautiously. Future longitudinal studies should use objective device-specific measurements and assess viewing distance, break frequency, continuous screen use, outdoor exposure and baseline refractive status to better determine whether digital behaviours independently contribute to childhood myopia.

### 3.3. Outdoor Activity, Lifestyle Factors and Parental Myopia

Outdoor activity appeared to be both a protective factor and an important confounder in the included studies. Enthoven et al. and Sánchez-Tena et al. found that outdoor exposure appeared to reduce the negative effect of near work on myopia risk [16,28]. This was supported by Alvarez-Peregrina et al., who found that myopic children had lower outdoor activity than non-myopic peers [22]. The Madrid study by Nieves-Moreno et al. further supported this pattern, finding that outdoor activity, particularly during weekends, was associated with lower myopia prevalence, while screen time was not independently associated with myopia after multivariate adjustment [29]. Irish studies also reported that higher physical activity was associated with reduced myopia risk [23,24]. Together, these findings suggest that outdoor exposure and physical activity may reduce myopia risk and should be considered when interpreting studies of childhood visual behaviour.

The protective effect of outdoor activity is biologically plausible. Natural light exposure is thought to stimulate retinal dopamine release, which may inhibit excessive axial elongation and help regulate ocular growth [10,30,31,32]. This supports the idea that outdoor activity is not simply a confounding variable, but a potentially modifiable protective behaviour. Therefore, children with lower outdoor exposure may be at higher risk, particularly when combined with other visually demanding behaviours such as prolonged near work.

The COVID-19 studies further support the importance of environmental and lifestyle change. The Spanish study reported a significant myopic shift after home confinement, with mean spherical equivalent changing from +0.66 ± 2.03 D in 2019 to +0.48 ± 1.81 D in 2020 (*p* ≤ 0.001) [26]. This was supported by Trovato Battagliola’s Italian study which reported significant myopia prevalence and refractive outcomes among children aged 5–12 years after lockdown [18]. These findings are consistent with wider evidence of accelerated myopia-related outcomes during confinement periods [13,33]. However, the value of these studies is not in isolating one specific exposure, but in showing that refractive outcomes may worsen when children’s routines shift towards more indoor activity, reduced outdoor exposure and altered daily behaviour.

Parental myopia was another important factor because it may influence risk through both genetic and behavioural pathways. A Portuguese study showed myopia prevalence of 12.7% among 1992 children and found that parental myopia increased its risk [28]. Iyer et al. also showed that parents with myopia had greater myopia awareness and were more likely to reduce their children’s near-screen use [25]. This suggests that parental myopia may influence not only biological susceptibility, but also parental attitudes, monitoring and prevention behaviours [34].

Broader epidemiological studies provide useful context for these findings. Schuster et al. reported increasing myopia prevalence among 17,640 children and adolescents, highlighting the growing public health importance of childhood myopia [19]. Rudnicka et al. similarly described variation and increasing trends in childhood myopia prevalence, with environmental factors such as near work and reduced outdoor exposure implicated as contributors [27]. Although these studies do not directly assess individual lifestyle behaviours in detail, they support the wider interpretation that childhood myopia is multifactorial and influenced by both environmental and familial factors.

Overall, outdoor activity, physical activity and parental myopia are important when interpreting myopia risk, as they reflect the interaction between environment, lifestyle and genetic susceptibility [10,30,31,35]. Studies that do not account for these factors may overestimate the independent effect of near work. Prevention should therefore focus on increasing outdoor time, promoting breaks from near work and identifying children at higher familial risk.

## 4. Discussion

Taken together, the evidence suggests that screen-related behaviours are associated with childhood myopia in European populations, but the relationship is complex, heterogeneous and likely multifactorial. Screen exposure should not be interpreted as a single independent causal exposure, but rather as part of a broader visual and behavioural environment that may include prolonged near work, close viewing distance, limited breaks, reduced outdoor exposure, indoor lifestyle patterns and genetic susceptibility. This interpretation aligns with international evidence linking near work, digital device use and reduced outdoor exposure with myopia [8,11,14]. However, the certainty of evidence in this review was low to very low, and causal conclusions cannot be drawn.

Recent systematic reviews and meta-analyses have examined myopia prevalence and digital device exposure more broadly. For example, Martinez-Perez et al. recently analysed myopia prevalence across European populations, while other reviews have focused on digital smart device use [14] or global risk factors for myopia [27,34]. However, these reviews either focused primarily on prevalence, included non-European populations, or considered broader age groups and exposure definitions. In contrast, the present review specifically synthesises evidence on screen-related behaviours and myopia-related outcomes in European children and adolescents. This regional and exposure-focused approach is intended to complement, rather than replace, wider international evidence [36].

The findings have practical implications for paediatric eye health, but recommendations should be framed cautiously. Since digital devices are now embedded in education and daily life, advice focused solely on reducing screen time may be unrealistic and insufficient. A more balanced approach would promote regular outdoor activity, breaks from prolonged near work, appropriate viewing distances and avoidance of prolonged uninterrupted handheld device use. In European settings, this could support school-based education, parental guidance and early identification of children at higher risk, particularly those with parental myopia or early refractive change.

Several limitations should be considered. Although the search was expanded to include Ovid Embase, Ovid MEDLINE, Scopus, Web of Science and Cochrane Library/CENTRAL, the review was restricted to English-language studies, which may have introduced language bias. The review protocol was not prospectively registered, which reduces methodological transparency and should be acknowledged as a limitation. Restricting the review to European paediatric populations provided a region-specific synthesis but also narrowed the evidence base and limits external generalisability.

The included studies were also methodologically heterogeneous. Most were observational, and many were cross-sectional, limiting causal inference. Screen exposure was inconsistently defined, with studies assessing total screen time, smartphone use, internet data consumption, computer use, handheld near-screen use or broader near-work behaviour. Many studies relied on self- or parent-reported exposure data, increasing the risk of recall bias and misclassification. Adjustment for confounders, including outdoor activity, parental myopia, socioeconomic status, educational intensity and baseline refractive status, was inconsistent. Therefore, the findings should be interpreted cautiously as evidence of association rather than causation.

Future research should use standardised screen exposure definitions and separate handheld near-screen use, computer use, television viewing and educational screen use. Objective measures of screen duration, viewing distance, break frequency and outdoor exposure would improve accuracy. Longitudinal studies using cycloplegic refraction and axial length measurements are needed to determine whether digital device use independently contributes to myopia onset or progression.

## 5. Conclusions

This systematic review identified observational evidence suggesting an association between screen-related behaviours and myopia-related outcomes in European children and adolescents. However, the available evidence is heterogeneous and of low to very low certainty, with most included studies being observational and many relying on self- or parent-reported measures of screen exposure. Consequently, the current evidence does not support causal conclusions or allow firm comparisons between different screen modalities.

The findings suggest that screen-related behaviours should be considered alongside other established risk factors, including outdoor activity, parental myopia and broader near-work behaviours. Practical advice should therefore focus on promoting balanced digital device use, reducing prolonged uninterrupted near work and encouraging regular outdoor activity rather than concentrating on screen time alone.

Future longitudinal studies using standardised definitions of screen exposure, objective exposure measures, cycloplegic refraction and axial length outcomes are required to better understand the independent contribution of digital device use to childhood myopia.

## Figures and Tables

**Table 1:** Risk of bias assessment of cohort studies using the Newcastle–Ottawa Scale.

Study	Study Design	Selection	Comparability	Outcome/Exposure	Total NOS Score
Enthoven et al. (2020) [16]	Cohort study	4/4	1/2	3/3	8/9
Hansen et al. [17]	Cohort study	3/4	1/2	2/3	6/9
Trovato Battagliola et al. [18]	Cohort study	3/4	1/2	3/3	7/9

**Table 2:** Risk of bias assessment of cross-sectional studies using the Joanna Briggs Institute (JBI) analytical cross-sectional checklist.

Study	Criteria for Inclusion Clearly Defined	Detailed Description of Subjects and Setting	Exposure Measured in a Valid and Reliable Way	Condition Measured Using Objective and Standard Criteria	Confounding Factors Identified	Strategies to Deal with Confounding Factors Stated	Outcomes Measured in a Valid and Reliable Way	Appropriate Statistical Analysis	Overall Risk of Bias
Schuster et al. [19]	Yes	Yes	Unclear	No	Yes	Yes	Unclear	Yes	Moderate
Mccrann et al. [20]	Unclear	Unclear	Yes	Yes	Unclear	Unclear	Yes	Unclear	Moderate
Enthoven et al. (2021) [21]	Yes	Yes	Yes	Yes	Yes	Yes	Yes	Yes	Low
Alvarez-Peregrina et al. (2020) [22]	Yes	Yes	Unclear	Yes	Yes	Yes	Yes	Yes	Moderate
Harrington et al. [23]	Yes	Yes	Unclear	Yes	Yes	Yes	Yes	Yes	Low
Harrington & O’dwyer [24]	Yes	Yes	Unclear	Yes	Yes	Yes	Yes	Yes	Low
Iyer et al. [25]	Yes	Yes	Yes	N/a	Yes	Yes	Yes	Yes	Moderate
Alvarez-Peregrina et al. (2021) [26]	Yes	Yes	Unclear	Yes	Yes	No	Yes	Yes	Moderate
Rudnicka et al. [27]	Yes	Yes	Unclear	N/a	Yes	Yes	Yes	Yes	Moderate
Sánchez-Tena et al. [28]	Yes	Yes	Unclear	Yes	Yes	Yes	Yes	Yes	Moderate
Nieves-Moreno et al. [29]	Yes	Yes	No	Yes	Yes	Yes	Yes	Yes	Moderate

**Table 3:** Summary of the characteristics of the included studies, including design, country, sample size, age group, exposure, key confounders considered or adjusted for, and main findings.

Author	Year	Country	Study Design	Sample Size	Age Group	Exposure	Key Confounders Considered/Adjusted for	Main Findings
Schuster et al. [19]	2020	Germany	Cross-sectional	17,640	Children/adolescents	Time trends	Age, sex, time period/survey wave	Myopia prevalence increased
McCrann et al. [20]	2021	Ireland	Cross-sectional	402	Children	Smartphones	Age, sex, refractive status; proxy smartphone data use	Myopes used more smartphone data than non-myopes (1130.7 vs. 613.6 MB/day), suggesting a positive association.
Enthoven et al. [21]	2021	Netherlands	Cross-sectional	525	Adolescents	Smartphones	Age, sex, outdoor exposure, continuous smartphone use	Smartphone use of ≥20 min continuously was linked with more myopic refraction, especially with low outdoor time.
Alvarez-Peregrina et al. [22]	2020	Spain	Cross-sectional	7497	Children	Screens/outdoor time	Age, sex, outdoor time, screen time	Myopia prevalence was 19%; myopic children had more screen time and less outdoor time (*p* < 0.01), when compared to non-myopic children
Enthoven et al. [16]	2020	Netherlands	Cohort	5074	Children	Computer use	Age, sex, parental myopia, outdoor exposure, near work	Myopia prevalence was 11.5%; computer use and near work increased risk (OR 1.005–1.072).
Harrington et al. [23]	2019	Ireland	Cross-sectional	1626	Children	Near-work/lifestyle	Age, sex, physical activity, screen time, reading/near work	Over half of myopic children exceeded 2 h/day screen time, compared with approximately one-third of the overall population.
Harrington & O’Dwyer [24]	2023	Ireland	Cross-sectional	723	Children	Screens/reading	Age, sex, screen time, reading/near work, physical activity	Higher screen time was linked with more myopic refraction and increased odds of myopia
Iyer et al. [25]	2025	Netherlands	Cross-sectional	395	Children	Handheld screens	Parental myopia, parental awareness, screen-use behaviours	26.0% used near screens for >2 h/day; myopic parents showed greater myopia awareness and were more likely to reduce children’s screen time.
Alvarez-Peregrina et al. [26]	2021	Spain	Cross-sectional	703	Children	COVID confinement	Age, screen time, near work, outdoor time, confinement-related behaviour	After COVID confinement, mean refraction shifted from +0.66 D to +0.48 D, with more near work and less outdoor time.
Hansen et al. [17]	2020	Denmark	Cohort	1442	Adolescents	Screens/activity	Age, sex, physical activity, screen-device use	Myopia prevalence was about 25%; >6 h/day screen use roughly doubled myopia risk.
Trovato Battagliola et al. [18]	2021	Italy	Cohort	180	Children	COVID lockdown	Age, lockdown-related lifestyle change, refractive status	Children showed a significant myopic shift after lockdown, suggesting worsening refractive error post-confinement.
Rudnicka AR et al. [27]	2016	UK	Cross-sectional	Multiple Studies	Children	Near-work/outdoor	Age, sex, ethnicity/geographical region, time trends	Childhood myopia varied globally and increased over time, with near work and low outdoor time implicated.
Sánchez-Tena et al. [28]	2024	Portugal	Cross-sectional	1992	Children	Parental myopia, outdoor activity and lifestyle factors	Parental myopia, outdoor activity, lifestyle factors	Myopia prevalence was 12.7%; parental myopia increased risk, while outdoor activity was protective.
Nieves-Moreno et al. [29]	2025	Spain	Cross-sectional	2489	Children	Screen time, outdoor activity, parental myopia	Age/grade, sex, parental myopia, outdoor activity, near work, screen time, socioeconomic status	Myopia prevalence was 6.5% in second grade and 18.7% in sixth grade. Parental myopia was a significant risk factor, outdoor activity was protective, and screen time was not independently associated with myopia after multivariate adjustment.

## Data Availability

No new data were created or analysed in this study.

## Supplementary Materials

The following supporting information can be downloaded at https://www.mdpi.com/article/10.3390/vision10030043/s1, Table S1: Completed PRISMA 2020 checklist [15].

## Abbreviations

The following abbreviations are used in this manuscript:

AI	Artificial Intelligence
PRISMA	Preferred Reporting Items for Systematic Reviews and Meta-Analyses
PICO	Population, Intervention/Exposure, Comparison, Outcome
D	Dioptres
IMI	International Myopia Institute
WHO	World Health Organisation
COVID-19	Coronavirus Disease 2019
NOS	Newcastle–Ottawa Scale
JBI	Joanna Briggs Institute
OR	Odds Ratio
CI	Confidence Interval
MB/day	Megabytes Per Day
UK	United Kingdom
n	Number/Sample Size
*p*	*p*-Value/Statistical Significance Value
N/a	Not Applicable
OVID MEDLINE	Ovid MEDLINE Database
OVID Embase	Ovid Embase Database
GRADE	Grading of Recommendations Assessment, Development and Evaluation
CENTRAL	Cochrane Central Register of Controlled Trials
SE	Spherical Equivalent

## Appendix A

**Table A1:** PICO table.

PICO Element	Description
P (Population)	Children and adolescents (<18 years) living in European countries
I (Intervention/Exposure)	Digital screen exposure, including screen time, smartphone use, tablet use, computer use, and other digital device use
C (Comparison)	Lower screen exposure, reduced digital device use, or no reported exposure
O (Outcome)	Development or progression of myopia, including refractive error (spherical equivalent), myopia prevalence/incidence, axial elongation, or worsening visual outcomes

## Appendix B

**Table A2:** Full Search strategy.

Database	Date of Search	Search Terms	Filters	Results
OVID Embase	January 2026	(myop* OR near-sightedness OR short-sightedness OR “refractive error”) AND (“screen time” OR smartphone* OR tablet* OR “digital device*” OR “computer use”) AND (adolescent* OR teenager* OR schoolchildren OR child* OR paediatric OR pediatric)	Language: English; publication years: 2016–2026 Age < 18	425
OVID MEDLINE	January 2026	(myop* OR near-sightedness OR short-sightedness OR “refractive error”) AND (“screen time” OR smartphone* OR tablet* OR “digital device*” OR “computer use”) AND (adolescent* OR teenager* OR schoolchildren OR child* OR paediatric OR pediatric)	Language: English; publication years: 2016–2026 Age < 18	228
Scopus	July 2026	TITLE-ABS-KEY (myop* OR “near-sightedness” OR “short-sightedness” OR “refractive error”) AND TITLE-ABS-KEY (“screen time” OR smartphone* OR tablet* OR “digital device*” OR “computer use”) AND TITLE-ABS-KEY (adolescent* OR teenager* OR schoolchildren OR child* OR paediatric OR pediatric)	English language; publication years 2016–2026; article	282
Web of Science	July 2026	TS = (myop* OR “near-sightedness” OR “short-sightedness” OR “refractive error”) AND TS = (“screen time” OR smartphone* OR tablet* OR “digital device*” OR “computer use”) AND TS = (adolescent* OR teenager* OR schoolchildren OR child* OR paediatric OR pediatric)	English language; publication years 2016–2026; article	219
Cochrane Library/CENTRAL	July 2026	(myop* OR “near-sightedness” OR “short-sightedness” OR “refractive error”) AND (“screen time” OR smartphone* OR tablet* OR “digital device*” OR “computer use”) AND (adolescent* OR teenager* OR schoolchildren OR child* OR paediatric OR paediatric)	Publication years 2016–2026; CENTRAL/trials	42

Note: The asterisk (*) was used as a truncation symbol to retrieve all terms sharing the same word root but with different suffixes.

## References

[1] Flitcroft D.I., He M., Jonas J.B., Jong M., Naidoo K., Ohno-Matsui K., Rahi J., Resnikoff S., Vitale S., Yannuzzi L. (2019). IMI—Defining and Classifying Myopia: A Proposed Set of Standards for Clinical and Epidemiologic Studies. Investig. Ophthalmol. Vis. Sci..

[2] Fredrick D.R. (2002). Myopia. BMJ.

[3] Saw S.M., Gazzard G., Shih-Yen E.C., Chua W. (2005). Myopia and Associated Pathological Complications. Ophthalmic Physiol. Opt..

[4] Flitcroft D.I. (2012). The Complex Interactions of. Retinal, Optical and Environmental Factors in Myopia Aetiology. Prog. Retin. Eye Res..

[5] Pan W., Morgan I.G., Flitcroft I., Rose K., Ostrin L.A., Rosenfield M., Govender-Poonsamy P., Siu-Villaseñor D., Kaymak H., Khew J.M. (2025). The Need to Address the Myopia Pandemic: Summary Report of the Global Myopia Public. Health Summit 2024. Glob. Health Res. Policy.

[6] Lanca C., Saw S.M. (2020). The Association between Digital Screen Time and Myopia: A Systematic Review. Ophthalmic Physiol. Opt..

[7] Holden B.A., Fricke T.R., Wilson D.A., Jong M., Naidoo K.S., Sankaridurg P., Wong T.Y., Naduvilath T., Resnikoff S. (2016). Global Prevalence of Myopia and High Myopia and Temporal Trends from 2000 through 2050. Ophthalmology.

[8] Saxena R., Vashist P., Tandon R., Pandey R.M., Bhardawaj A., Menon V., Mani K. (2015). Prevalence of Myopia and Its Risk Factors in Urban School Children in Delhi: The North India Myopia Study (NIM Study). PLoS ONE.

[9] Morgan I.G., Ohno-Matsui K., Saw S.M. (2012). Myopia. Lancet.

[10] Rose K.A., Morgan I.G., Ip J., Kifley A., Huynh S., Smith W., Mitchell P. (2008). Outdoor Activity Reduces the Prevalence of Myopia in Children. Ophthalmology.

[11] Huang H.M., Chang D.S., Wu P.C. (2015). The Association between Near Work Activities and Myopia in Children. PLoS ONE.

[12] Tideman J.W.L., Polling J.R., Jaddoe V.W., Vingerling J.R., Klaver C.C. (2019). Environmental Risk Factors Can Reduce Axial Length Elongation and Myopia Incidence in 6- to 9-Year-Old Children. Ophthalmology.

[13] Wang J., Li Y., Musch D.C., Wei N., Qi X., Ding G., Li X., Li J., Song L., Zhang Y. (2021). Progression of Myopia in School-Aged Children after COVID-19 Home Confinement. JAMA Ophthalmol..

[14] Foreman J., Salim A.T., Praveen A., Fonseka D., Ting D.S.W., He M.G., Bourne R.R.A., Crowston J., Wong T.Y., Dirani M. (2021). Association between Digital Smart Device Use and Myopia: A Systematic Review and Meta-Analysis. Lancet Digit. Health.

[15] Page M.J., McKenzie J.E., Bossuyt P.M., Boutron I., Hoffmann T.C., Mulrow C.D., Shamseer L., Tetzlaff J.M., Akl E.A., Brennan S.E. (2021). The PRISMA 2020 Statement: An Updated Guideline for Reporting Systematic Reviews. BMJ.

[16] Enthoven C.A., Tideman J.W.L., Polling J.R., Yang-Huang J., Raat H., Klaver C.C.W. (2020). The Impact of Computer Use on Myopia Development in Childhood: The Generation R Study. Prev. Med..

[17] Hansen M.H., Laigaard P.P., Olsen E.M., Skovgaard A.M., Larsen M., Kessel L., Munch I.C. (2020). Low Physical Activity and Higher Use of Screen Devices Are Associated with Myopia. Acta Ophthalmol..

[18] Trovato Battagliola E., Mangiantini P., D’Andrea M., Malvasi M., Loffredo L., Scalinci S.Z., Comberiati A.M., Migliorini R., Pacella E. (2023). Effect of COVID-19 Lockdown on Refractive Errors in Italian Children Aged 5–12 Years: A Multi-Center Retrospective Study. Eur. J. Ophthalmol..

[19] Schuster A.K., Krause L., Kuchenbaecker C., Prütz F., Elflein H.M., Pfeiffer N., Urschitz M.S. (2020). Prevalence and Time Trends in Myopia among Children and Adolescents: Results of the German KiGGS Study. Dtsch. Arztebl. Int..

[20] McCrann S., Loughman J., Butler J.S., Paudel N., Flitcroft D.I. (2021). Smartphone Use as a Possible Risk Factor for Myopia. Clin. Exp. Optom..

[21] Enthoven C.A., Polling J.R., Verzijden T., Tideman J.W.L., Al-Jaffar N., Jansen P.W., Raat H., Metz L., Verhoeven V.J.M., Klaver C.C.W. (2021). Smartphone Use Associated with Refractive Error in Teenagers: The Myopia App Study. Ophthalmology.

[22] Alvarez-Peregrina C., Sánchez-Tena M.A., Martinez-Perez C., Villa-Collar C. (2020). The Relationship between Screen and Outdoor Time with Rates of Myopia in Spanish Children. Front. Public Health.

[23] Harrington S.C., Stack J., O’Dwyer V. (2019). Risk Factors Associated with Myopia in Schoolchildren in Ireland. Br. J. Ophthalmol..

[24] Harrington S.C., O’Dwyer V. (2023). The Association between Time Spent on Screens and Reading with Myopia, Premyopia and Ocular Biometric and Anthropometric Measures in 6- to 7-Year-Old Schoolchildren in Ireland. Ophthalmic Physiol. Opt..

[25] Iyer V., Hermans R., Polling J.R., Klaver C., Reijneveld S. (2025). Parental Behavior and Near Screen Use in Childhood: A Route to Reduce Screen Induced Myopia. Front. Public Health.

[26] Alvarez-Peregrina C., Martínez-Pérez C., Villa-Collar C., Andreu-Vázquez C., Ruiz-Pomeda A., Sánchez-Tena M.Á. (2021). Impact of COVID-19 Home Confinement in Children’s Refractive Errors. Int. J. Environ. Res. Public Health.

[27] Rudnicka A.R., Kapetanakis V.V., Wathern A.K., Logan N.S., Gilmartin B., Whincup P.H., Cook D.G., Owen C.G. (2016). Global Variations and Time Trends in the Prevalence of Childhood Myopia: A Systematic Review and Quantitative Meta-Analysis. Br. J. Ophthalmol..

[28] Sánchez-Tena M.Á., Martínez-Pérez C., Andreu-Vázquez C., Roque A., Alvarez-Peregrina C. (2025). Factors Associated with Myopia in the Portuguese Child Population: An Epidemiological Study. Ophthalmic Physiol. Opt..

[29] Nieves-Moreno M., Carracedo-Rodriguez G., Piñero-Llorens D.P., Valderas L.B., Recalde-Maestre S., García-Da-Silva J., Díaz-Vega B., Llorente-Gonzalez S., Alarcón-Tomás M., Lovera-Rivas M. (2025). Prevalence and Risk Factors for Myopia in Primary School Children in Madrid: A School-Based Cycloplegic Refraction Study. Int. J. Environ. Res. Public Health.

[30] He M., Xiang F., Zeng Y., Mai J., Chen Q., Zhang J., Smith W., Rose K., Morgan I.G. (2015). Effect of Time Spent Outdoors at School on the Development of Myopia among Children in China: A Randomized Clinical Trial. JAMA.

[31] Wu P.-C., Tsai C.-L., Wu H.-L., Yang Y.-H., Kuo H.-K. (2013). Outdoor Activity during Class Recess Reduces Myopia Onset and Progression in School Children. Ophthalmology.

[32] Feldkaemper M., Schaeffel F. (2013). An Updated View on the Role of Dopamine in Myopia. Exp. Eye Res..

[33] Xu L., Ma Y., Yuan J., Zhang Y., Wang H., Zhang G., Tu C., Lu X., Li J., Xiong Y. (2021). COVID-19 Quarantine Reveals That Behavioral Changes Have an Effect on Myopia Progression. Ophthalmology.

[34] Pan C.W., Ramamurthy D., Saw S.M. (2012). Worldwide Prevalence and Risk Factors for Myopia. Ophthalmic Physiol. Opt..

[35] French A.N., Ashby R.S., Morgan I.G., Rose K.A. (2013). Time Outdoors and the Prevention of Myopia. Exp. Eye Res..

[36] Martinez-Perez C., Alvarez-Peregrina C., Villa-Collar C., Sánchez-Tena M.Á. (2026). Analysing Myopia in Europe: A Comprehensive Meta-Analysis. Graefe’s Arch. Clin. Exp. Ophthalmol..

