# Supplementary material for: Screen Time and Myopia-Related Outcomes in European Children: A Systematic Review
Source: Vision (Basel). 2026 Jul 13;10(3):43. doi: 10.3390/vision10030043 (PMC13398318; doi:10.3390/vision10030043)
Supplement: Supplementary file 1 [file vision-10-00043-s001.zip › vision-4409801-supplementary.pdf]

# Supplementary Materials: Screen Time and Myopia-Related Outcomes in European Children: A Systematic Review.

Hammaad Khalid, Ali Istanaksai and Mishelle Abbasi

Table S1: Completed PRISMA 2020 Main Checklist for Screen Time and Myopia-Related Outcomes in European Children: A Systematic Review

| Topic | No. | Item | Location where item is reported |
| --- | --- | --- | --- |
| **TITLE** | | | |
| **Title** | 1 | Identify the report as a systematic review. | Page 1, Title |
| **ABSTRACT** | | | |
| **Abstract** | 2 | | Abstract |
| **INTRODUCTION** | | | |
| **Rationale** | 3 | Describe the rationale for the review in the context of existing knowledge. | Page 2, Introduction |
| **Objectives** | 4 | Provide an explicit statement of the objective(s) or question(s) the review addresses. | Page 2, final paragraph of Introduction |
| **METHODS** | | | |
| **Eligibility criteria** | 5 | Specify the inclusion and exclusion criteria for the review and how studies were grouped for the syntheses. | Methods: Eligibility Criteria; Appendix Table A.1: PICO table |
| **Information sources** | 6 | Specify all databases, registers, websites, organisations, reference lists and other sources searched or consulted to identify studies. Specify the date when each source was last searched or consulted. | Methods: Search Strategy; Appendix Table B.1 : Search strategy |
| **Search strategy** | 7 | Present the full search strategies for all databases, registers and websites, including any filters and limits used. | Methods: Search Strategy; Appendix Table B.1 : Search strategy |

| Topic | No. | Item | Location where item is reported |
|---|---|---|---|
| **Selection process** | 8 | Specify the methods used to decide whether a study met the inclusion criteria of the review, including how many reviewers screened each record and each report retrieved, whether they worked independently, and if applicable, details of automation tools used in the process. | Methods: Study Selection; Figure 1: PRISMA Flow Diagram |
| **Data collection process** | 9 | Specify the methods used to collect data from reports, including how many reviewers collected data from each report, whether they worked independently, any processes for obtaining or confirming data from study investigators, and if applicable, details of automation tools used in the process. | Methods: Data Extraction |
| **Data items** | 10a | List and define all outcomes for which data were sought. Specify whether all results that were compatible with each outcome domain in each study were sought (e.g. for all measures, time points, analyses), and if not, the methods used to decide which results to collect. | Methods: Eligibility Criteria; Methods: Data Extraction; Appendix Table A.1: PICO table |
| | 10b | List and define all other variables for which data were sought (e.g. participant and intervention characteristics, funding sources). Describe any assumptions made about any missing or unclear information. | Methods: Data Extraction; Table 3: Study characteristics |
| **Study risk of bias assessment** | 11 | Specify the methods used to assess risk of bias in the included studies, including details of the tool(s) used, how many reviewers assessed each study and whether they worked independently, and if applicable, details of automation tools used in the process. | Methods: Risk of Bias Assessment; Table 1: Newcastle-Ottawa Scale; Table 2: Joanna Briggs Institute checklist |
| **Effect measures** | 12 | Specify for each outcome the effect measure(s) (e.g. risk ratio, mean difference) used in the synthesis or presentation of results. | Methods: Data Extraction; Methods: Data Synthesis. Effect estimates, p-values and refractive change measures were extracted where available; no pooled effect measure was calculated because meta-analysis was not performed |

| Topic | No. | Item | Location where item is reported |
| --- | --- | --- | --- |
| **Synthesis methods** | 13a | Describe the processes used to decide which studies were eligible for each synthesis (e.g. tabulating the study intervention characteristics and comparing against the planned groups for each synthesis (item 5)). | Methods: Data Synthesis |
| | 13b | Describe any methods required to prepare the data for presentation or synthesis, such as handling of missing summary statistics, or data conversions. | Methods: Data Synthesis. Data were extracted and summarised narratively due to heterogeneity |
| | 13c | Describe any methods used to tabulate or visually display results of individual studies and syntheses. | Table 3: Study characteristics and findings; Review section |
| | 13d | Describe any methods used to synthesize results and provide a rationale for the choice(s). If meta-analysis was performed, describe the model(s), method(s) to identify the presence and extent of statistical heterogeneity, and software package(s) used. | Methods: Data Synthesis. Meta-analysis was not performed; findings were synthesised narratively |
| | 13e | Describe any methods used to explore possible causes of heterogeneity among study results (e.g. subgroup analysis, meta-regression). | Methods: Data Synthesis; Discussion. Heterogeneity was considered narratively across study design, exposure definitions, outcome measures and confounder adjustment |
| | 13f | Describe any sensitivity analyses conducted to assess robustness of the synthesized results. | Methods: Data Synthesis. Not applicable; sensitivity analyses were not performed because meta-analysis was not undertaken |
| **Reporting bias assessment** | 14 | Describe any methods used to assess risk of bias due to missing results in a synthesis (arising from reporting biases). | Methods: Data Synthesis. Formal reporting bias assessment was not undertaken because of heterogeneity in study design and outcomes |
| **Certainty assessment** | 15 | Describe any methods used to assess certainty (or confidence) in the body of evidence for an outcome. | Methods: Data Synthesis. Formal certainty assessment was not undertaken because of heterogeneity in included study designs and outcomes |
| **RESULTS** | | | |

| Topic | No. | Item | Location where item is reported |
|---|---|---|---|
| **Study selection** | 16a | Describe the results of the search and selection process, from the number of records identified in the search to the number of studies included in the review, ideally using a flow diagram. | Figure 1: PRISMA Flow Diagram; Methods: Study Selection |
| | 16b | Cite studies that might appear to meet the inclusion criteria, but which were excluded, and explain why they were excluded. | Figure 1: PRISMA Flow Diagram. Reasons for full-text exclusion are reported as non-relevant and non-European population |
| **Study characteristics** | 17 | Cite each included study and present its characteristics. | Table 3: Summary of study characteristics |
| **Risk of bias in studies** | 18 | Present assessments of risk of bias for each included study. | Table 1: Risk of bias assessment of cohort studies; Table 2: Risk of bias assessment of cross-sectional studies |
| **Results of individual studies** | 19 | For all outcomes, present, for each study: (a) summary statistics for each group (where appropriate) and (b) an effect estimate and its precision (e.g. confidence/credible interval), ideally using structured tables or plots. | Table 3: Summary of study characteristics and findings; Review section |
| **Results of syntheses** | 20a | For each synthesis, briefly summarise the characteristics and risk of bias among contributing studies. | Tables 1–3; Review section |
| | 20b | Present results of all statistical syntheses conducted. If meta-analysis was done, present for each the summary estimate and its precision (e.g. confidence/credible interval) and measures of statistical heterogeneity. If comparing groups, describe the direction of the effect. | Review section: Screen Exposure and Myopia; Device Type and Near-Work; Outdoor Activity, Lifestyle Factors and Parental Myopia |
| | 20c | Present results of all investigations of possible causes of heterogeneity among study results. | Review section; Discussion. Heterogeneity was discussed narratively rather than statistically |
| | 20d | Present results of all sensitivity analyses conducted to assess the robustness of the synthesized results. | Not applicable. Sensitivity analyses were not performed because meta-analysis was not undertaken |
| **Reporting biases** | 21 | Present assessments of risk of bias due to missing results (arising from reporting biases) for each synthesis assessed. | Methods: Data Synthesis; Discussion. Formal reporting bias assessment was not undertaken due to heterogeneity |

| Topic | No. | Item | Location where item is reported |
|---|---|---|---|
| **Certainty of evidence** | 22 | Present assessments of certainty (or confidence) in the body of evidence for each outcome assessed. | Methods: Data Synthesis; Discussion. Formal certainty assessment was not undertaken due to heterogeneity |
| **DISCUSSION** | | | |
| **Discussion** | 23a | Provide a general interpretation of the results in the context of other evidence. | Discussion |
| | 23b | Discuss any limitations of the evidence included in the review. | Discussion, limitations paragraph |
| | 23c | Discuss any limitations of the review processes used. | Discussion, limitations paragraph, including restriction to two databases, English-language studies and observational evidence |
| | 23d | Discuss implications of the results for practice, policy, and future research. | Discussion; Conclusions |
| **OTHER INFORMATION** | | | |
| **Registration and protocol** | 24a | Provide registration information for the review, including register name and registration number, or state that the review was not registered. | Methods, first paragraph: “The review protocol was not prospectively registered.” |
| | 24b | Indicate where the review protocol can be accessed, or state that a protocol was not prepared. | Methods, first paragraph. Not applicable; no review protocol was prospectively registered or published |
| | 24c | Describe and explain any amendments to information provided at registration or in the protocol. | Not applicable; no registered or published protocol was available |
| **Support** | 25 | Describe sources of financial or non-financial support for the review, and the role of the funders or sponsors in the review. | Funding statement; This research received no external funding. |
| **Competing interests** | 26 | Declare any competing interests of review authors. | Conflicts of Interest statement; The authors declare no conflict of interest. |

| Topic | No. | Item | Location where item is reported |
| --- | --- | --- | --- |
| **Availability of data, code and other materials** | 27 | Report which of the following are publicly available and where they can be found: template data collection forms; data extracted from included studies; data used for all analyses; analytic code; any other materials used in the review. | All data generated or analysed during this review are included in the manuscript and appendices. The completed PRISMA 2020 checklist is provided as Supplementary Table S1 |

*From:* Page MJ, McKenzie JE, Bossuyt PM, Boutron I, Hoffmann TC, Mulrow CD, et al. The PRISMA 2020 statement: an updated guideline for reporting systematic reviews. *BMJ* **2021**, *372*, n71. https://doi.org/10.1136/bmj.n71. For more information, visit: www.prisma-statement.org
